# Simultaneous extraction of caffeic acid and production of cellulose microfibrils from coffee grounds using hydrodynamic cavitation in a Venturi tube

**DOI:** 10.1016/j.ultsonch.2025.107370

**Published:** 2025-04-23

**Authors:** Hitoshi Soyama, Kousuke Hiromori, Naomi Shibasaki-Kitakawa

**Affiliations:** a Department of Finemechanics, Tohoku University, 6-6-01 Aramaki, Aoba-ku, Sendai 980-8579, Japan; b Department of Chemical Engineering, Tohoku University, 6-6-07 Aramaki, Aoba-ku, Sendai 980-8579, Japan

**Keywords:** Hydrodynamic cavitation, Cellulose microfibril, Caffeic acid, Biomass, Coffee grounds

## Abstract

Large quantities of spent coffee grounds (SCGs) are produced daily across the globe, accumulating as industrial waste in factories. Developing a process that both extracts high-value components and utilizes the bulk material would offer significant academic and industrial advantages. This study explores the use of hydrodynamic cavitation, which utilizes the chemical and physical effects produced by bubble collapse, for high-efficiency, continuous processing. The optimization of cavitation conditions was conducted by measuring the aggressive intensity of hydrodynamic cavitation within a Venturi tube. Then, unbrewed coffee grounds was processed by hydrodynamic cavitation to obtain stable results, as caffeic acid in SCGs varied depending on how the coffee was brewed. It was revealed that the hydrodynamic cavitation in the Venturi tube increased extraction rate of coffeic acid and simultaneously generates cellulose microfibrils. Note that the upstream pressure of the Venturi tube was 3.4 MPa, which was generated by a screw pump, and the aggressive intensity of the hydrodynamic cavitation was enhanced by optimizing the downstream pressure of the Venturi tube. The type of cavitation, closely linked to the aggressive intensity, was also analyzed through high-speed photography.

## Graphical abstract

**Figure:**
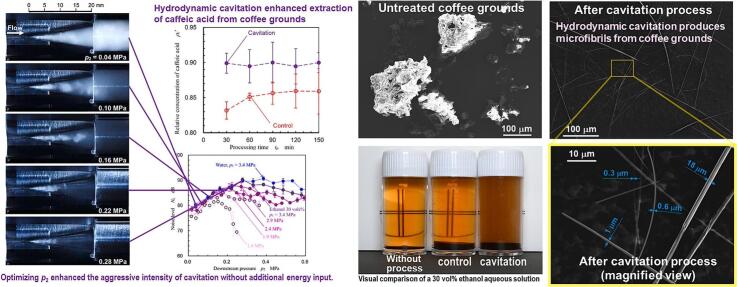

## 1 Introduction

According to a report by the International Coffee Organization, global coffee consumption is projected to reach approximately 171.3 million 60 kg bags in the 2022/2023 period [1]. There is a growing need for sustainable applications of spent coffee grounds (SCGs), which have accumulated as substantial industrial waste [[2], [3], [4]]. Proposals for extracting biologically active substances such as polyphenols [[5], [6], [7], [8]] and producing biofuel [9] have been put forward, although the primary constituents of SCGs remain largely untapped as waste. Additionally, while methods to enhance the biodegradability of SCGs have been suggested [10], the overall cost of SCG treatment needs consideration. Caffeic acid, which can be extracted from SCGs, has potential applications in cancer treatment [[11], [12], [13], [14], [15], [16], [17]] and the conversion of SCG's main composition into nanofibers or microfibrils for industrial use suggests a sustainable and valuable process, transforming a vast amount of waste into useful industrial materials that produce valuable agents.

Cavitation bubble collapse generates significant physical impacts [18] and chemical effects, including high-temperature and high-pressure zones [19]. These cavitation effects can improve the fatigue strength of metallic materials [[20], [21], [22]] and have been explored for extracting high-value ingredients from renewable biomass [23]. Hydrodynamic cavitation, known for its utility in water treatment [[24], [25], [26], [27], [28], [29]], also shows promise in applications ranging from selective removal of pathogens like Escherichia coli and daphnia [30] to food processing [31], treatment of food wastes [32], wastewater management [24,33,34]. Moreover, it facilitates the degradation of harmful chemicals such as bisphenol A [35,36] and estrogen [37], and aids in the removal of contaminants like succinic acid [38], and dyes [39,40]. Additionally, hydrodynamic cavitation is beneficial in the degradation of ammonia nitrogen [41], biofouling removal [42], synthesis of methyl esters [43,44], and production of ethanol and xylitol [45], among other applications. It is also utilized in oxygen injection [46], preparation of magnesium hydroxide [47], intensification of biogas production [48,49], biodiesel generation [[50], [51], [52], [53]], production of nanoparticles [54], extraction of soybean proteins [55], lemon pectin [56], and proteins and carbohydrates from macroalgae [57], as well as in the pretreatment of biomass [[58], [59], [60], [61], [62], [63], [64], [65], [66], [67], [68], [69], [70], [71], [72], [73]].

When considering the primary composition of SCGs, they can be transformed into cellulose nanofibers or microfibrils [[74], [75], [76]]. These nanocellulose materials are valuable for developing polymer matrix composites [77,78], high-performance electromagnetic interference (EMI) shielding materials [79], and sustainable active packaging [80]. The mechanical properties of cellulose nanofibers, such as tensile strength, have been extensively studied to facilitate their application in industrial materials [81]. Regarding the production methods, techniques like ultrasonication [[82], [83], [84], [85], [86]] and microfluidic device-based cavitation [87] have been proposed. Notably, the pretreatment efficiency of hydrodynamic cavitation has been reported to be 20 times more effective than ultrasonic cavitation [58], suggesting that further exploration of cellulose nanofiber and microfibril production via hydrodynamic cavitation is warranted.

In terms of generating hydrodynamic cavitation for chemical reactors [88], various configurations have been explored, including cavitating jets [89], Venturi tubes [58,90], orifice plates [91,92], jet pumps [93,94], swirling vortex flows [[95], [96], [97]], rotor-radial grooves [98], and rotor–stator designs [52]. It has been observed that vortex cavitation in a Venturi tube is particularly effective, strongly correlated with the aggressive intensity of hydrodynamic cavitation [99]. This study focuses on the application of hydrodynamic cavitation within a Venturi tube, examining its efficiency in detail. The relationship between cavitation types—such as spherical bubbles, swirling, tip, and turbulent vortex cavitation—and the aggressive intensity of cavitation collapse is crucial, as the turbulent eddies in the Venturi tube intensify the cavitation collapse [100]. While the extraction of bioactive substances like caffeic acid from SCGs is promising, the sustainability of the process is challenged by the large volume of residue generated. However, converting this residue into industrially valuable materials like cellulose nanofibers or microfibrils could render the process both economically and environmentally beneficial.

In this study, a method for extracting caffeic acid from unbrewed coffee grounds using hydrodynamic cavitation in a Venturi tube was investigated, aiming for more consistent results while concurrently converting the residue into cellulose microfibrils. The simultaneous extraction of caffeic acid and production of cellulose microfibrils were validated experimentally. As the aggressive intensity of cavitation varies with the type of cavitation as mentioned above, the aspect of hydrodynamic cavitation was observed by high speed photography.

## 2 Materials and methods

### 2.1 Materials

To achieve a more stable extraction rate of caffeic acid from coffee grounds, unbrewed coffee grounds were selected due to the variability in caffeic acid content in SCGs based on the brewing method. The coffee grounds used were from a consistent lot of commercially available paper filter drip coffee (Home Brewed Product Series, Kilimanjaro Blend, KEY COFFEE INC, Tokyo, Japan). These grounds were pulverized in a blender and sifted through a wire mesh with a 355 µm aperture. In the extraction process, 15 g of coffee grounds were mixed into 3 L of a 30 vol% ethanol aqueous solution. This particular concentration of ethanol was chosen because it effectively dissolves caffeic acid while aligning with the requirements for managing the aggressive intensity of the hydrodynamic cavitation. The aggressive intensity was evaluated by measuring the luminescence intensity generated by hydrodynamic cavitation in a Venturi tube, as noted in a previous study [99] and by evaluating the cavitation erosion rate using a cavitating jet apparatus [101]. The experimental setup involved an injection pressure of 0.5 MPa for luminescence intensity measurements and 10 MPa for cavitation erosion testing. The main findings, illustrating the variation of the luminescence intensity and cavitation erosion rate with the ethanol concentration, are detailed in Appendices A and B. Both the low and high injection pressure tests showed peak aggressive intensities at a 30 vol% ethanol concentration, which was subsequently used in the experiments.

### 2.2 Hydrodynamic cavitation apparatus using Venturi tube

**Fig. 1:**
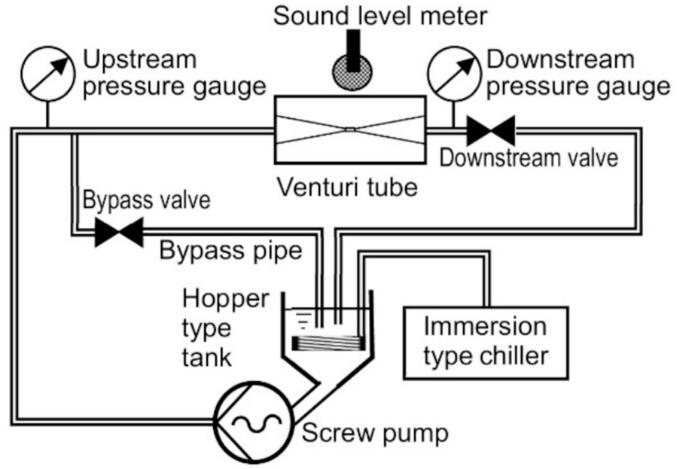
Schematic diagram of experimental setup.

**Fig. 2:**
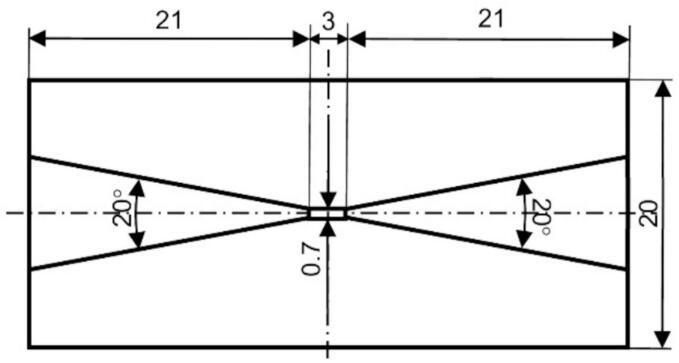
Schematic of the Venturi tube (unit: mm).

Fig. 1, Fig. 2 provide a schematic of hydrodynamic cavitation using a Venturi tube and detail its geometric specifications. The throat diameter of the Venturi tube measures 0.7 mm. Both the inlet and outlet angles are set at 20°, as depicted in Fig. 2. The tube is constructed from brass for operational durability and acrylic resin to facilitate observation of the cavitation process. A 30 vol% ethanol aqueous solution containing coffee grounds is loaded into a hopper-type tank, which holds up to 6.6 L and has a maximum diameter of 210 mm. This solution is pressurized by a screw pump capable of reaching a maximum pressure of 3.6 MPa and a flow rate of 3.8 L/min. The tank and pump are interconnected using a stainless steel pipe with an inner diameter of 35 mm, while the connecting pipes for the pump, valves, and Venturi tube have an inner diameter of 8 mm. During the cavitation process, a bypass valve is closed to ensure the solution is pressurized by the pump and directed into the Venturi tube. The upstream pressure of the Venturi tube, denoted as *p*_1_, is regulated by adjusting the pump's rotational speed. Conversely, the downstream pressure (*p*_2_) is modified by manipulating the downstream valve. Both upstream and downstream pressures are monitored using pressure gauges. In the control process, the downstream valve is closed, allowing the pressurized solution to be recirculated back into the tank via a bypass pipe with an 8 mm inner diameter. The pump's rotational speed during the control process is maintained at the same level as during the cavitation condition.

The cavitation number, *σ*, a critical parameter in cavitating flows [18], is defined by Eq. (1), as *p*_1_ and *p*_2_ were described as gauge pressures in MPa.

(1) $$\sigma =\frac{\left(p_{2}+0.1\right)-p_{v}}{{(p}_{1}+0.1)-\left(p_{2}+0.1\right)}=\frac{\left(p_{2}+0.1\right)-p_{v}}{p_{1}-p_{2}},$$

where *p_v_* is the vapor pressure of the liquid. In this study, *p_v_* was calculated from the vapor pressures of water and ethanol considering the concentration ratio of ethanol. During the process, the solution temperature was kept at 293 ± 2 K by an immersion type chiller.

### 2.3 Evaluation of aggressive intensity of hydrodynamic cavitation

It has been observed that the aggressive intensities of acoustic noise and luminescence generated by hydrodynamic cavitation in the Venturi tube vary with *p*_1_ and *p*_2_ [99,102]. These aggressive intensities were measured using a sound level meter, which can assess cavitation noise [103]. The meter was positioned perpendicular to the Venturi tube, at a distance of 50 mm. For the measurement, Z-weighted sound levels, which remain flat from 10 Hz to 20 kHz, were employed. The noise level (*N_L_*) was recorded as an average over 10 s and expressed in decibels (dB). Since the acoustic intensity is proportional to the square of the acoustic pressure (*p_a_*) [103], the aggressive intensity of cavitation (*I_cav_*) was defined by Eq. (2), where *p_a_* is derived from the *N_L_*.

(2) $$I_{\mathrm{cav}}=\;\frac{\;{p_{a}}^{2}\;}{\rho \;c}\;=\frac{\;{\left(\;20\;\times \;10^{-6}\;\times \;10^{\frac{\;\;N_{L}\;}{\;20}}\;\;\right)}^{2}\;}{\rho \;c},$$

where *ρ* and *c* denote the density and velocity of sound in the solution, respectively. The pump power *P_J_* [kW] for upstream pressure *p*_1_ [MPa] and flow rate *Q* [L/min] are defined by Eq. (3), the treatment efficiency of coffee grounds using cavitation was considered. Note that *Q*, which varied with *p*_2_ at constant *p*_1_ of used the Venturi tube, was approximately equivalent as shown in Appendix C.

(3) $$P_{J}=\frac{1\times 10^{6}\times p_{1}\times Q}{60\eta },$$

where *η* is efficiency of the pump.

**Fig. 3:**
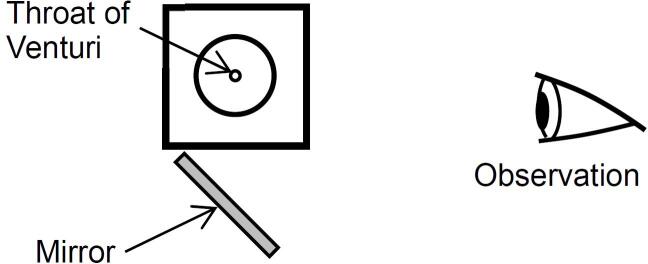
Diagram showing the direction of observation.

The aggressive intensity of cavitation, closely linked to bubble geometry [100], was meticulously observed using a high-speed video camera capable of capturing up to 8,000 frames per second (fps) in full frame (1,600 × 1,200 pixels) and up to 194,000 fps in partial frame (1,600 × 16 pixels). Additionally, a digital camera with a resolution of 5,568 × 3,712 pixels, equipped with a flash lamp, was used to capture detailed images. The flash lamp's exposure time was set to 1.5 μs. Both high-speed video and digital cameras were positioned perpendicularly to the Venturi tube. To enhance the visibility of the cavitation process, observations from two different angles were facilitated using a mirror, as depicted in Fig. 3.

### 2.4 Characterization of treated coffee grounds

To demonstrate the production of microfibrils from coffee grounds, the solution and sediment were freeze-dried and examined under a scanning electron microscope (SEM).

The concentration of extracted caffeic acid was determined using high-performance liquid chromatography (HPLC) system equipped with an ultraviolet detector (Waters Corp. Milford, MA. USA, ACQUITY UPLC H-Class System) and an ACQUITY UPLC BEH C18 column (Waters Corp., particle size 1.7 μm, i.d. 2.1 mm, length150 mm). Acetonitrile (Wako Pure Chemical Industries, Ltd., Osaka, Japan, HPLC AR Grade), trifluoroacetic acid (Wako Pure Chemical Industries, Ltd., HPLC AR grade), and ultrapure water were used as a mobile phase at a flow rate of 0.6 cm^3^/min by a gradient elution. The temperature of the column oven was 40 °C and the detection wavelength was 325 nm. In this study, the extraction rate was quantified relative to the benchmark extraction rate of caffeic acid using the Soxhlet extraction method.

## 3 Results

### 3.1 Aggressive intensity of hydrodynamic cavitation in Venturi tube

**Fig. 4:**
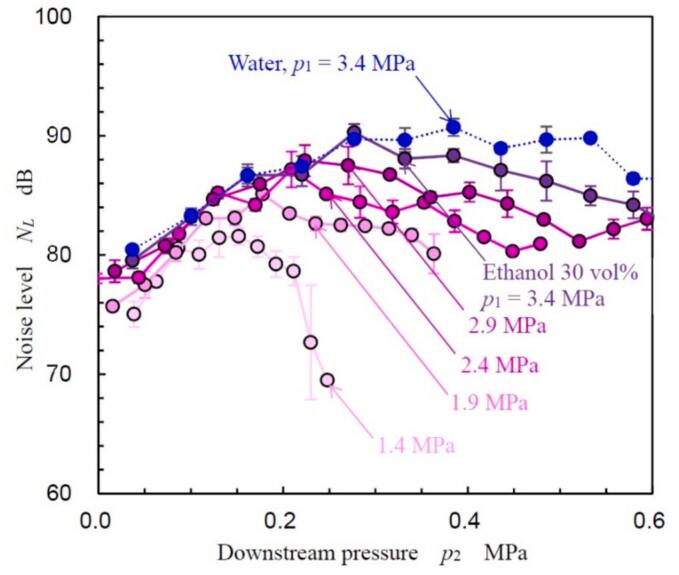
Variation in noise level (*N_L_*) with downstream pressure (*p*_2_).

Fig. 4 shows the noise level *N_L_* of the cavitation noise generated by the hydrodynamic cavitation of 30 vol% ethanol aqueous solution measured by the sound level meter as a function of *p*_2_ at *p*_1_ = 1.4, 1.9, 2.4, 2.9 and 3.4 MPa. As the sound level was measured three times at each condition, the plot in Fig. 4 shows mean value and error bar reveals standard deviation. The noise level *N_L_* owing to the hydrodynamic cavitation of water at *p*_1_ = 3.4 MPa is shown in Fig. 4. All *N_L_* values increased with increasing *p*_2_, peaked, and then decreased. As discussed in section “*3.2. Aspect of hydrodynamic cavitation in Venturi tube*”, the cavitating region decreased monotonically with an increase in *p*_2_, however, *N_L_* had a peak with respect to *p*_2_. This behavior of *N_L_* is consistent with patterns observed in acoustic energy [102] and luminescence intensity [99,102]. Notably, measuring *N_L_* with a sound level meter is considerably simpler than measuring acoustic energy via pulse height analysis [102] or luminescence intensity, as it does not require specialized equipment.

**Fig. 5:**
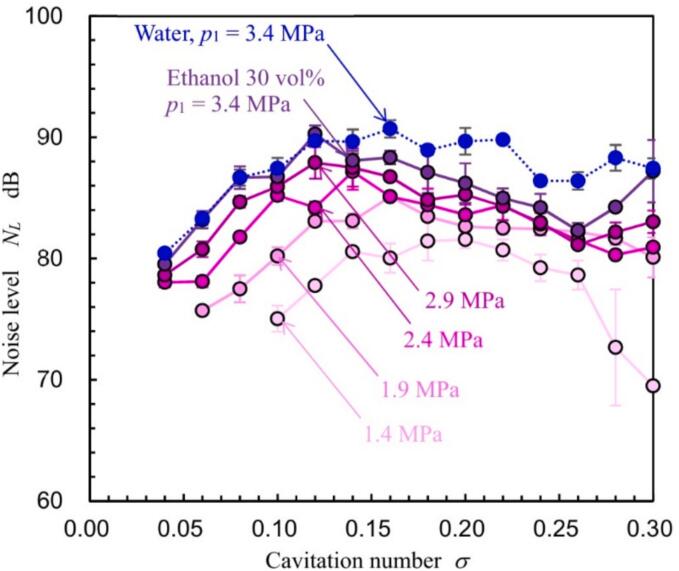
Noise level (*N_L_*) as a function of the cavitation number (*σ*).

To clarify the mechanism behind the peak in *N_L_* against *p*_2_, Fig. 5 illustrates *N_L_* as a function of the cavitation number *σ*. The plot and error bar in Fig. 5 show mean value and standard deviation as same as Fig. 4. As shown in Fig. 4, *p*_2_ at which *N_L_* had the maximum value, i.e., *N_Lmax_*, increased with *p*_1_. For example, *p*_2_ at which *N_L_* had maximum, *p*_2 max_, was 0.152 MPa for *p*_1_ = 1.4 MPa, 0.178 MPa for *p*_1_ = 1.9 MPa, 0.209 MPa for *p*_1_ = 2.4 MPa, 0.224 MPa for *p*_1_ = 2.9 MPa, and 0.28 MPa for *p*_1_ = 3.4 MPa. On the other hand, the peak *N_L_* occurred at *σ* = 0.12 – 0.16 for *p*_1_ = 1.9, 2.4, 2.9 and 3.4 MPa. This suggests that the tendency of *N_L_* to vary with *p*_2_ is a characteristic of a cavitating flow, similar to the main parameter *σ*. It has been demonstrated that the aggressive intensity of hydrodynamic cavitation in a Venturi tube is estimated by the sound velocity, frequency of vortex cavitation, and cavitating length [99]. Although the cavitation length decreases with *p*_2_, the sound velocity, which increases the individual cavitation bubble collapse, increases with *p*_2_ under a constant *p*_1_ condition [99].

**Fig. 6:**
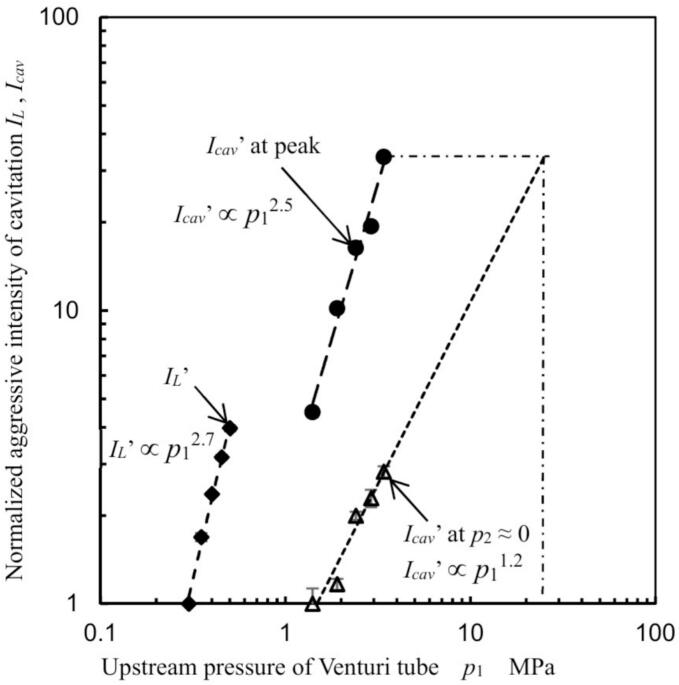
Variation in aggressive intensity of cavitation with upstream pressure in the Venturi tube.

**Fig. C1:**
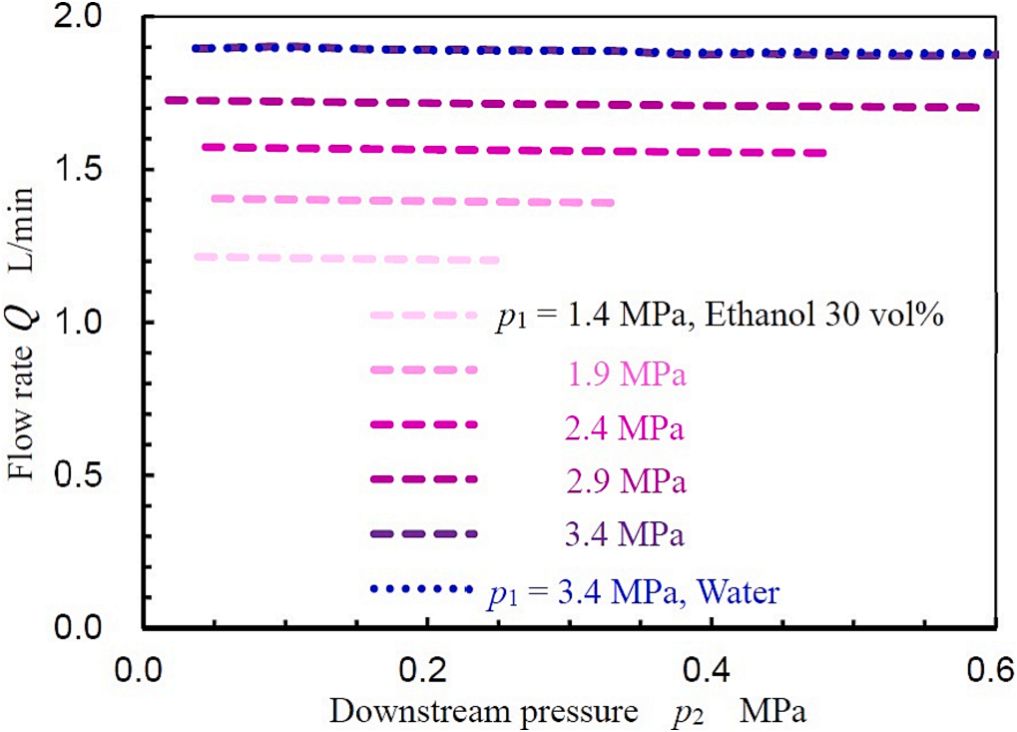
Flow rate changing with downstream pressure

To explore process efficiency improvements by optimizing *p*_2_, Fig. 6 depicts the normalized aggressive intensity *I_cav_*’ of hydrodynamic cavitation in the Venturi tube as a function of injection pressure *p*_1_. As *Q* at constat *p*_1_ was approximately equivalent as shown in Fig. C1 (Appendix C), *P_J_* was proportional to *p*_1_, then Fig. 6 reveals *I_cav_*’ as a function of *p*_1_. In Fig. 6, the aggressive intensity *I_cav_*, which was defined by Eq. (2), was normalized by *I_cav_* at *p*_1_ = 1.4 MPa and *p*_2_ = 0.06 MPa. As shown in Fig. 5, *N_L_* at *p*_2_ ≈ 0 MPa and *p*_2_ = *p*_2 max_ were increased with *p*_1_. Thus, *I_cav_*’ at *p*_2_ ≈ 0 MPa and *I_cav_*’ at *p*_2_ = *p*_2 max_ were increased. In Fig. 6, the luminescence intensity *I_L_* [99] at *p*_2_ = *p*_2 max_ was also revealed. *I_L_* was normalized to *I_L_* at *p*_1_ = 0.3 MPa. Assuming a power law relationship for the variation of *I_cav_*’ and *I_L_*’ with *p*_1_, the following equations were derived.

(4) $$I_{\mathrm{cav}}^{\prime }\propto {p_{1}}^{1.2}\;,\;\;\;\;\;\;\;\;\left(p_{2}\approx 0\;MPa\right),$$

(5) $$I_{\mathrm{cav}}^{\prime }\propto {p_{1}}^{2.5},\left(p_{2}=p_{2max}\right),$$

(6) $$I_{L}^{\prime }\propto {p_{1}}^{2.7},\left(p_{2}=p_{2max}\right).$$

As shown in Eqs. (4), (5), (6), the exponents at *p*_2_ = *p*_2 max_ are larger than those at *p*_2_ ≈ 0 MPa. Moreover, the value 2.5 from Eq. (5) closely approximates the 2.7 in Eq. (6). Fig. 6 demonstrates that *I_cav_*’ at *p*_1_ = 3.4 MPa and *p*_2_ = *p*_2 max_ is 7.4 times greater than that at *p*_1_ = 3.4 MPa and *p*_2_ ≈ 0. Assuming extrapolation as per Eq. (4), *I_cav_*’ at *p*_1_ = 3.4 MPa and *p*_2_ = *p*_2 max_ equates to *I_cav_*’ at *p*_1_ = 25 MPa and *p*_2_ ≈ 0 MPa. According to Eq. (3), the required electrical power for the pump at *p*_1_ = 25 MPa is 20 times greater than at *p*_1_ = 3.4 MPa. Therefore, optimizing *p*_2_ is advantageous for reducing pump power consumption. Reflecting the *N_L_* findings from Fig. 4, Fig. 5, the process conditions for the coffee grounds were set at *p*_1_ = 3.4 MPa and *p*_2_ = 0.28 MPa, where *N_L_* reaches its maximum in a 30 vol% ethanol aqueous solution.

### 3.2 Aspect of hydrodynamic cavitation in Venturi tube

**Fig. 7:**
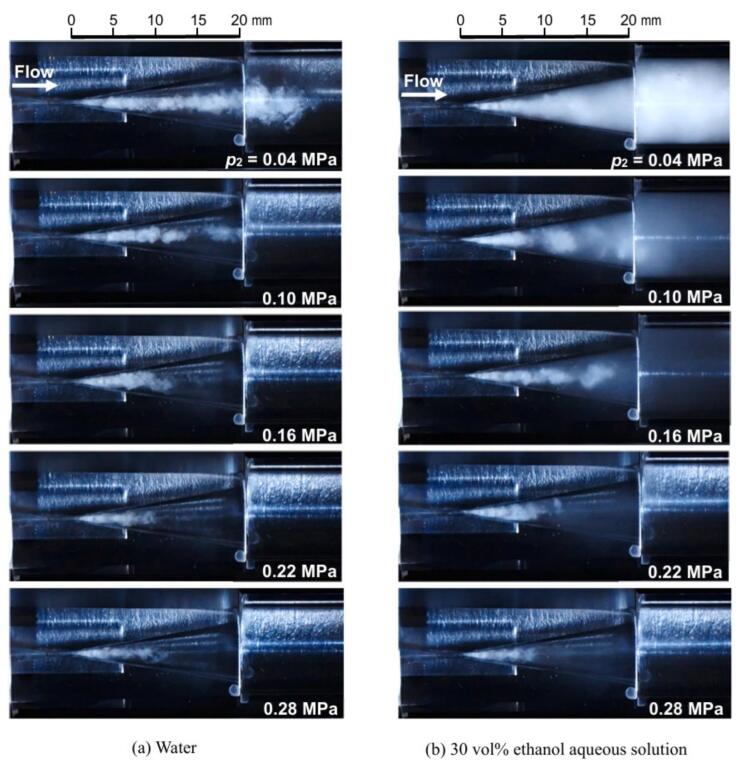
Visual representation of cavitation dynamics at different downstream pressures (*p*_2_) with upstream pressure *p*_1_ = 3.4 MPa:

Fig. 7 depicts changes in hydrodynamic cavitation at *p*_1_ = 3.4 MPa across (a) water and (b) 30 vol% ethanol aqueous solutions, with the flow direction from left to right. The white areas resembling clouds in Fig. 7 indicate the cavitating region. In both (a) water and (b) the ethanol solution, cavitation develops downstream from the throat, and the cavitating length decreases consistently with an increase in *p*_2_. Downstream vortex cavitation, characterized by angulated rather than spherical bubbles, was observed [104]. Despite the reduction in cavitating length from *p*_2_ = 0.04 MPa to *p*_2_ = 0.28 MPa, *N*_L_ increased. This increase may be attributed to the intensified individual cavitation collapses due to a decreased void ratio in the cavitation region, compounded by an increased acoustic impedance from higher sound velocities [99]. Comparing cavitation effects between water and the solution, the residual bubbles following cavitation collapse in the solution were notably denser at *p_2_* = 0.04 MPa, especially evident as the white regions in the solution appeared denser than those in water. The differences between water and the solution diminish with increasing *p*_2_, and the presence of vortex cavitation becomes more pronounced. In summary, the characteristic cavitation in a 30 vol% ethanol aqueous solution at *p*_1_ = 3.4 MPa and *p*_2_ = 0.28 MPa was identified as vortex cavitation, similar to that observed in water.

**Fig. 8:**
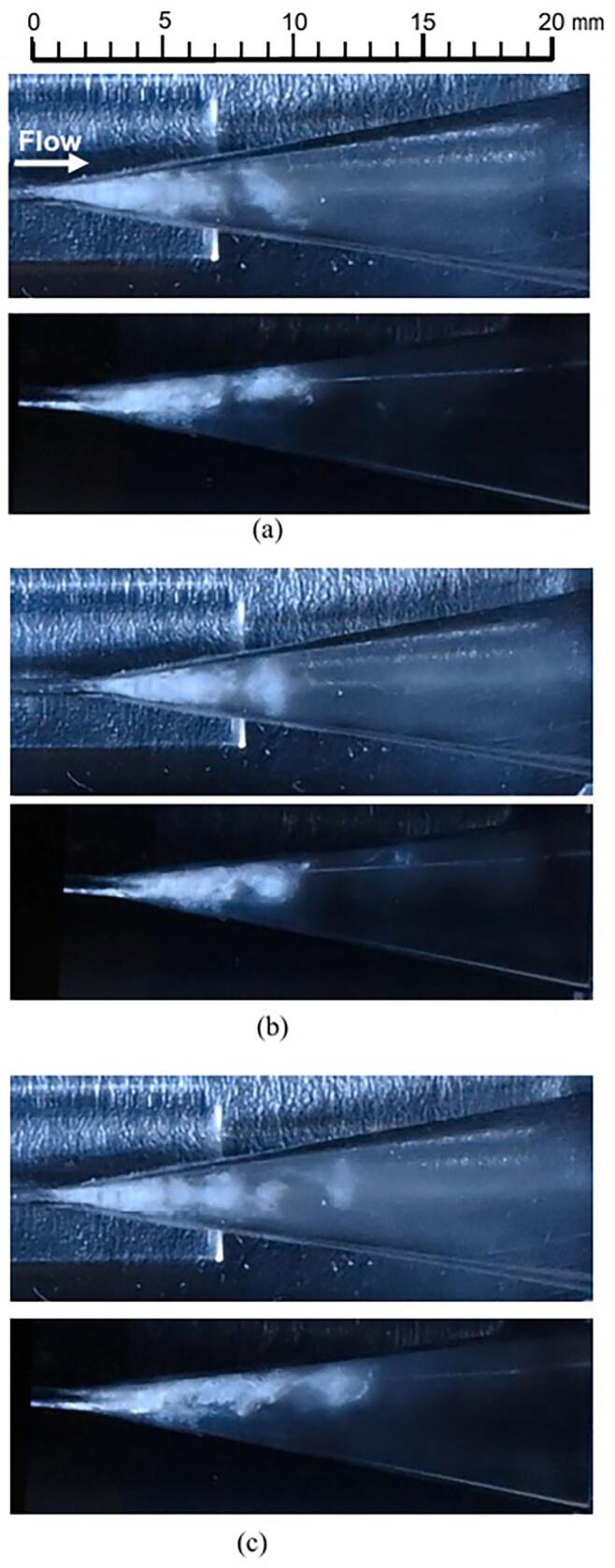
Visualization of vortex cavitation without coffee grounds observed from two perspectives in a 30 vol% ethanol aqueous solution (*p*_1_ = 3.4 MPa, *p*_2_ = 0.28 MPa).

**Fig. 9:**
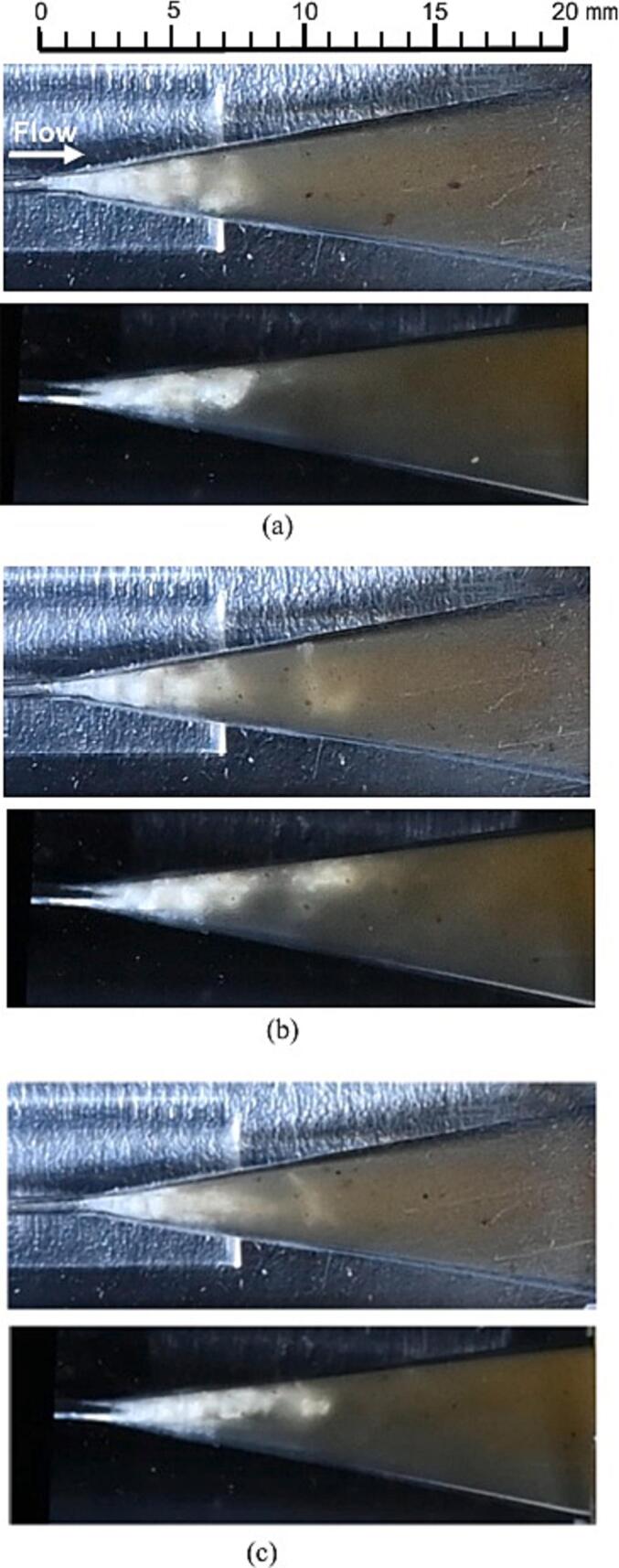
Visualization of vortex cavitation with coffee grounds observed from two perspectives in a 30 vol% ethanol aqueous solution (*p*_1_ = 3.4 MPa, *p*_2_ = 0.28 MPa).

To examine the structure of vortex cavitation, Fig. 8, Fig. 9 display the cavitation observed from two perspectives using a mirror (refer to Fig. 3), both with and without coffee grounds. The flow directions in Fig. 8, Fig. 9 are consistent with those in Fig. 7. Each of these figures illustrates three typical representations of vortex cavitation at the conditions of *p*_1_ = 3.4 MPa and *p*_2_ = 0.28 MPa. In each image set, the upper photo shows a direct view while the lower photo depicts the mirrored image. Due to the Venturi tube's design, which includes a corner at the throat inlet and outlet, the cavitating region appears slightly skewed, with the top part of the mirrored image exhibiting a longer cavitating region than the bottom part. As clearly demonstrated in Fig. 8 (a), the vortex cavitation located 10 mm from the throat outlet is inclined at a 45° angle relative to the main flow direction resembling the helical vortex structures previously reported [104]. Similarly, Fig. 9 (a), (b), and (c) show that vortex cavitation is also distinctly visible in the hydrodynamic cavitation of the solution.

**Fig. 10:**
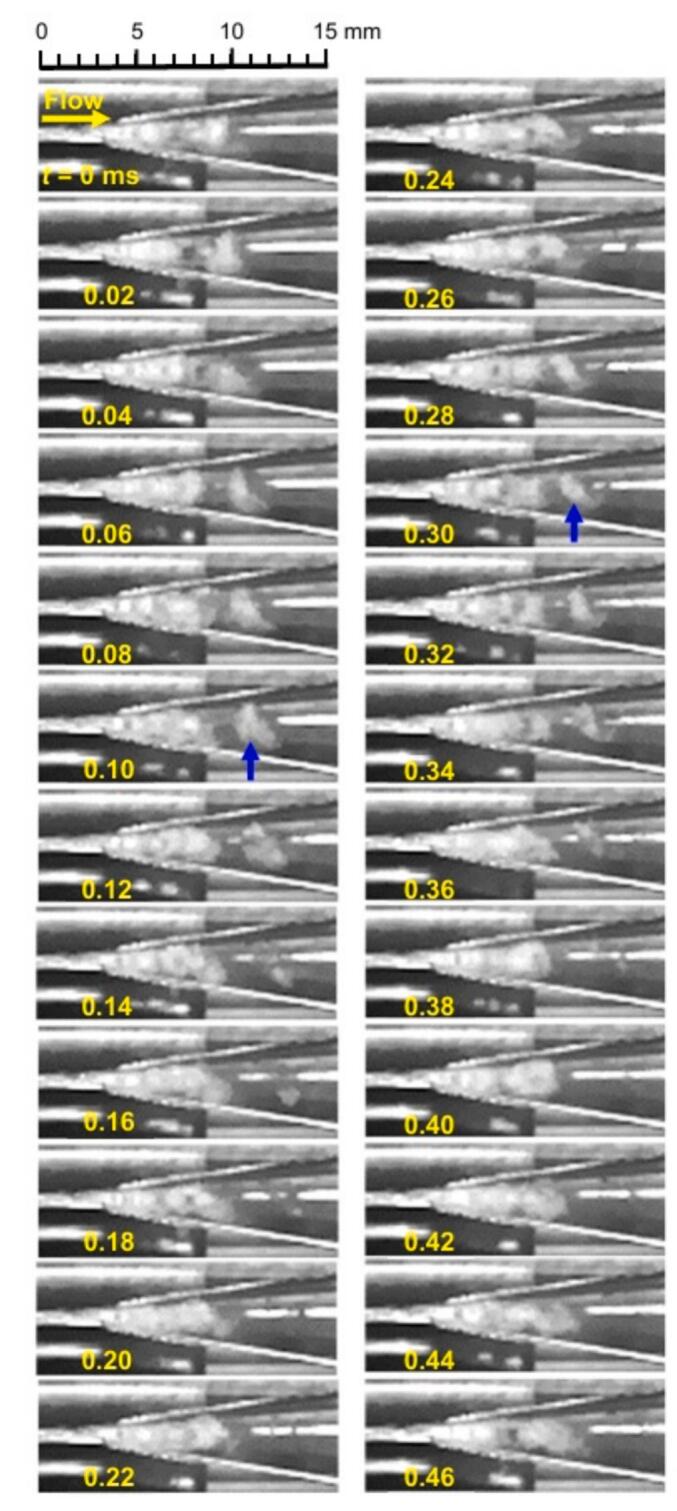
Vortex cavitation observed by high-speed video camera in a 30 vol% ethanol aqueous solution (*p*_1_ = 3.4 MPa, *p*_2_ = 0.28 MPa).

To further explore how vortex cavitation evolves over time, Fig. 10 presents high-speed video camera footage of hydrodynamic cavitation in a 30 vol% ethanol aqueous solution mixed with coffee grounds at *p*_1_ = 3.4 MPa and *p*_2_ = 0.28 MPa. The recording speed was set at 50,000 fps. In this figure, the flow direction aligns with that in Fig. 7, Fig. 8, Fig. 9. The vortex cavitation, indicated by blue arrows at *t* = 0.10 ms and *t* = 0.30 ms, is observed to be angled at 45° to the main flow direction. These vortex cavitations are shed from the trailing edges of the cavitating regions that develop from the throat and subsequently collapse, generating pressure during their collapse [99]. However, under the current conditions, it is very challenging to distinguish coffee grounds using high-speed video cameras.

### 3.3 Extraction of coffeic acid by hydrodynamic cavitation in Venturi tube

**Fig. 11:**
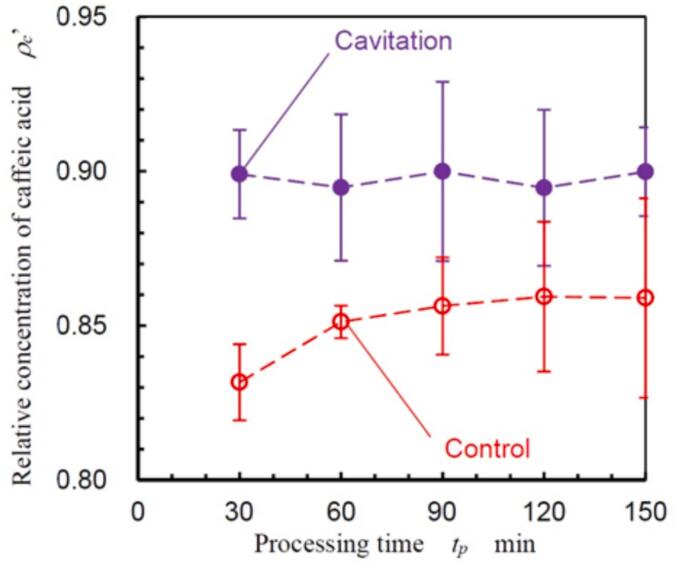
Variation of caffeic acid concentration as a function of processing time in a 30 vol% ethanol aqueous solution.

To demonstrate the effectiveness of extracting caffeic acid through hydrodynamic cavitation in a Venturi tube, Fig. 11 presents the relative concentration of caffeic acid (*ρ_c_*’) over the processing time (*t_p_*). The *ρ_c_*’ was determined by normalizing the caffeic acid concentration of non-treated coffee grounds measured by the Soxhlet extraction method, i.e., 6.544 × 10^-5^ mol/g. The conditions maintained were a *p*_1_ of 3.4 MPa and a *p*_2_ of 0.28 MPa, utilizing a 30 vol% ethanol aqueous solution. Samples of 15 ml were collected every 30 min. Both “control” and “cavitation” processes were conducted three times to ensure reliability. The circles in Fig. 11 show mean values, and the error bars indicate the standard deviations of three sets of experiments. At *t_p_* = 30 min, *ρ_c_*’ was 0.832 ± 0.012 for the control and 0.899 ± 0.014 for the cavitation process. Despite the standard deviation, there was a significant enhancement in extraction efficiency during cavitation. While *ρ_c_*’ for the control condition gradually increased and plateaued at 120 min, the cavitation process maintained *ρ_c_*’ approximately at 0.9 from 30 to 150 min, indicating that hydrodynamic cavitation under these specific conditions effectively enhanced the extraction of caffeic acid.

### 3.4 Production of cellulose microfibril by hydrodynamic cavitation in Venturi tube

**Fig. 12:**
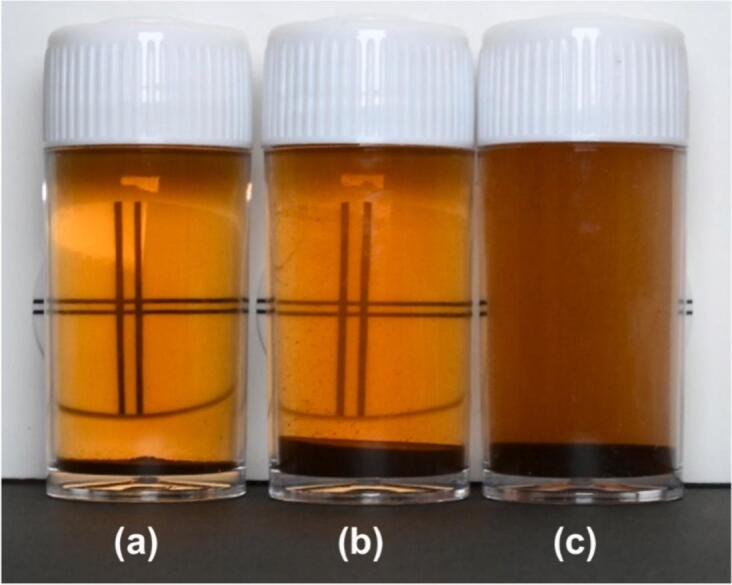
Visual comparison of a 30 vol% ethanol aqueous solution: (a) without processing, (b) after the control process for 150 min, (c) after the cavitation process for 150 min.

To illustrate the defibration of coffee grounds, Fig. 12 displays the appearance of 15 mL polystyrene bottles containing (a) the solution with coffee grounds prior to processing, (b) the solution with sediment after undergoing the control process for 150 min, and (c) the solution with sediment after experiencing cavitation for 150 min. Each scenario utilized a 30 vol% ethanol aqueous solution. Behind each bottle, a double-cross sign plate, employed in the transparency test for industrial wastewater according to the Japanese Industrial Standards JIS K0102-2021, was positioned. In the case of (a), 75 mg of coffee grounds were added to a 15 mL solution, maintaining a concentration of 0.5 wt%, consistent with the control and cavitation processes. In cases (b) and (c), after each 150-min process, the solutions were transferred into bottles with almost identical amounts of sediment and were left undisturbed for a week. As depicted in Fig. 12, the double-cross sign is clearly visible in (a) the untreated solution with coffee grounds, and (b) the solution post-control process. However, the sign is difficult to discern in (c) following the cavitation process, suggesting that cellulose microfibrils remained suspended in the solution in the cavitation case after 150 min.

**Fig. 13:**
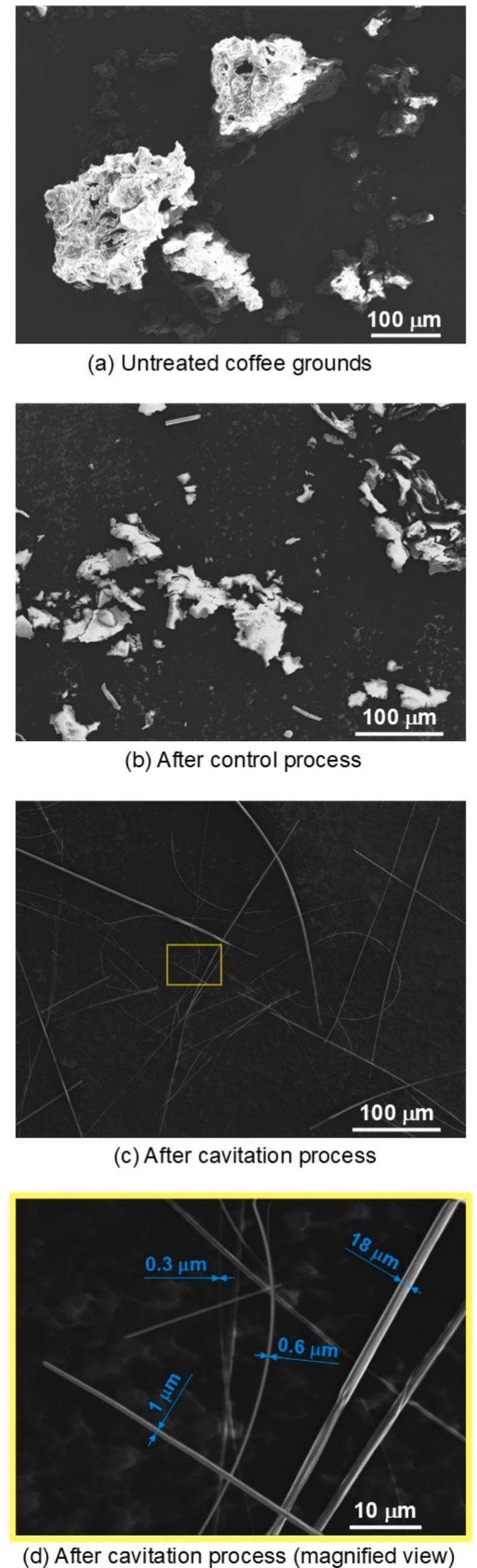
Scanning Electron Microscope (SEM) images of coffee grounds and microfibrils.

To compare the microfibrils produced with untreated coffee grounds, Fig. 13 displays: (a) untreated coffee grounds, (b) coffee grounds treated by the control process for 150 min, and (c) coffee grounds treated by cavitation for 150 min. For cases (b) and (c), after each respective process, coffee grounds were retrieved from the clear layer above the sediment and then freeze-dried. Specifically in case (c), the yellow rectangular area is enlarged in (d) to highlight details. In Fig. 13 (d), the diameters of several microfibrils are marked with blue annotations.

As depicted in Fig. 13 (a), the size of the untreated coffee grounds ranges from tens to hundreds of micrometers after being crushed and screened using a wire mesh. Fig. 13 (b) shows that the coffee grounds, though smaller than those in (a), still range from tens to hundreds of micrometers. In contrast, following the cavitation process, microfibrils ranging from submicrometers to several micrometers in diameter were produced, as shown in Fig. 13. The aspect ratio of these microfibrils exceeds 100, indicating that their length surpasses several hundred micrometers. Thus, it can be concluded that hydrodynamic cavitation in a Venturi tube under the conditions of *p*_1_ = 3.4 MPa and *p*_2_ = 0.28 MPa effectively transforms coffee grounds into microfibrils.

## 4 Conclusions

To develop a sustainable process for spent coffee grounds (SCGs), this study explored the use of hydrodynamic cavitation in a Venturi tube to simultaneously extract caffeic acid and produce cellulose microfibrils. The experimental setup involved a Venturi tube with a throat diameter of 0.7 mm, where the upstream pressure (*p*_1_) was maintained at 3.4 MPa. Unbrewed coffee grounds were chosen to ensure consistent results, as the ratio of caffeic acid in SCGs can vary with different brewing methods. The key findings from this study are summarized below:

1) Hydrodynamic cavitation, using a 30 vol% ethanol aqueous solution in the Venturi tube, effectively enhanced the extraction of caffeic acid from coffee grounds at *p*_1_ = 3.4 MPa and a downstream pressure (*p*_2_) of 0.28 MPa.
2) The process not only extracted caffeic acid but also simultaneously produced cellulose microfibrils.
3) The noise level of the cavitation, indicative of its aggressive intensity, peaked as a function of *p*_2_ at a constant *p*_1_. Optimizing *p*_2_ significantly enhanced the aggressive intensity of hydrodynamic cavitation without additional energy input. Specifically, optimizing the downstream pressure *p*_2_ of the Venturi tube reduced the electric power required for the pump by a factor of 20.
4) In experiments with a 30 vol% aqueous ethanol solution in the Venturi tube, a higher number of fine residual bubbles were observed at lower *p*_2_ values. At higher *p*_2_ values, these bubbles became less pronounced.
5) When hydrodynamic cavitation was performed with coffee grounds in a 30 vol% aqueous ethanol solution, vortex cavitation was clearly evident, similar to that observed with water. This vortex cavitation, inclined at a 45° angle to the main flow direction, was a component of the helical vortex.

These results demonstrate that hydrodynamic cavitation is a promising technique for valorizing spent coffee grounds by efficiently extracting valuable compounds and converting waste into useful byproducts.

## CRediT authorship contribution statement

**Hitoshi Soyama:** Writing – original draft, Visualization, Resources, Project administration, Methodology, Investigation, Funding acquisition, Formal analysis, Data curation, Conceptualization. **Kousuke Hiromori:** Writing – review & editing, Methodology, Investigation, Formal analysis, Data curation. **Naomi Shibasaki-Kitakawa:** Writing – review & editing, Supervision, Methodology, Conceptualization.

## Declaration of competing interest

The authors declare that they have no known competing financial interests or personal relationships that could have appeared to influence the work reported in this paper.

## Figures and Tables

**Fig. A1:**
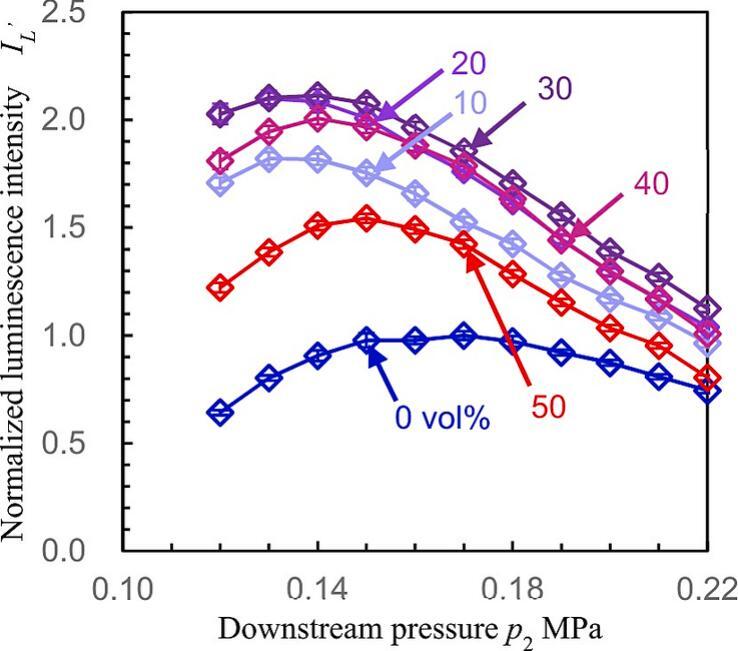
Luminescence intensity from hydrodynamic cavitation in the Venturi tube as a function of downstream pressure *p*_2_ across varying ethanol concentrations.

**Fig. A2:**
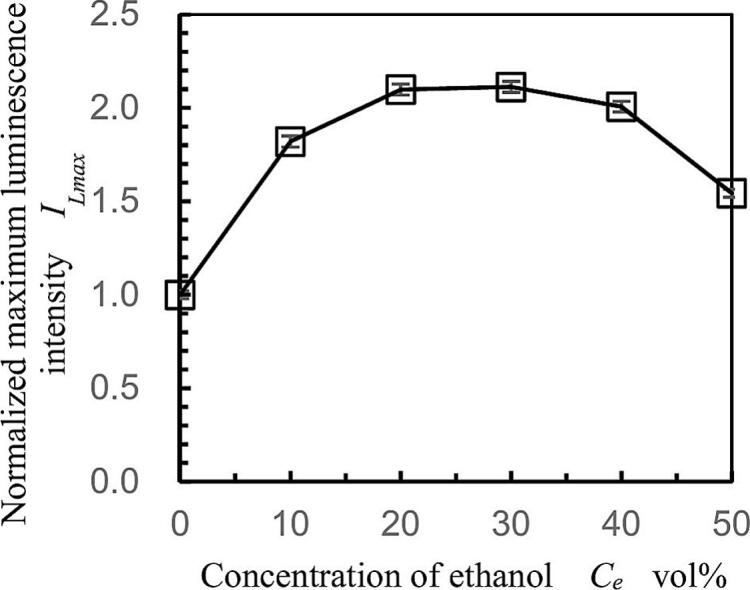
Maximum luminescence intensity from hydrodynamic cavitation in the Venturi tube across ethanol concentration gradients.

**Fig. B1:**
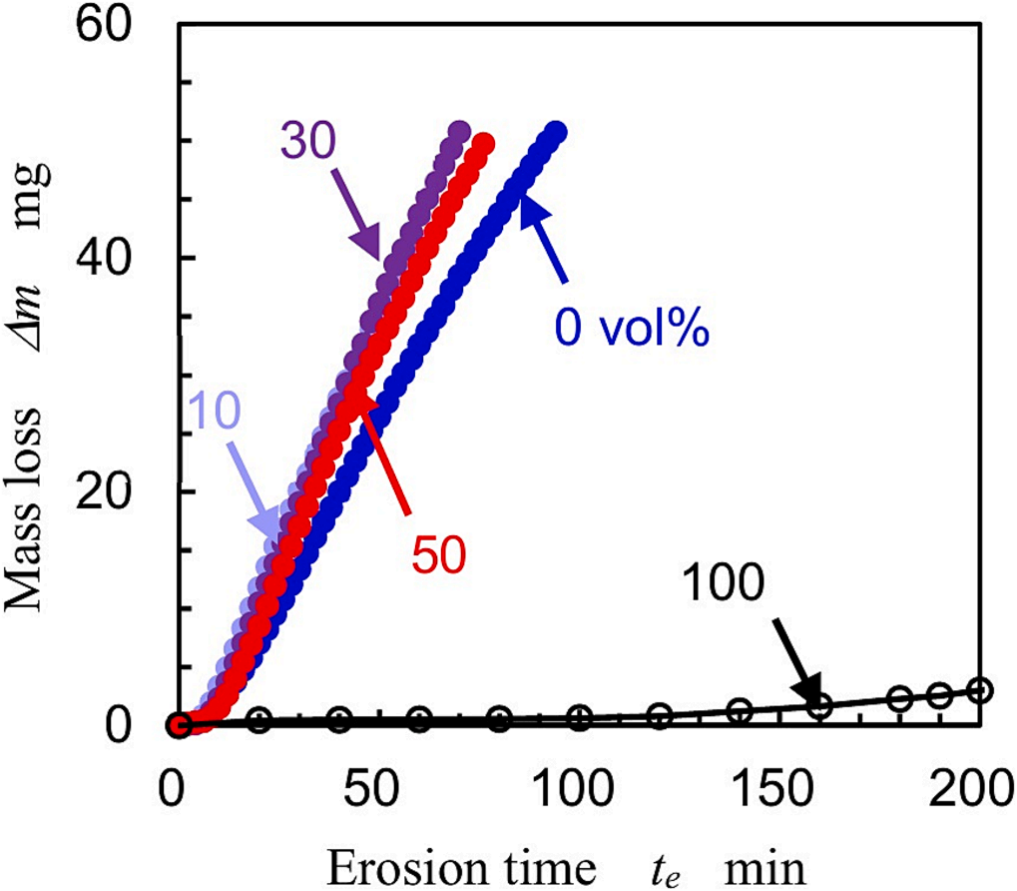
Mass loss over time during exposure to a cavitating jet, as per ASTM G134 apparatus.

**Fig. B2:**
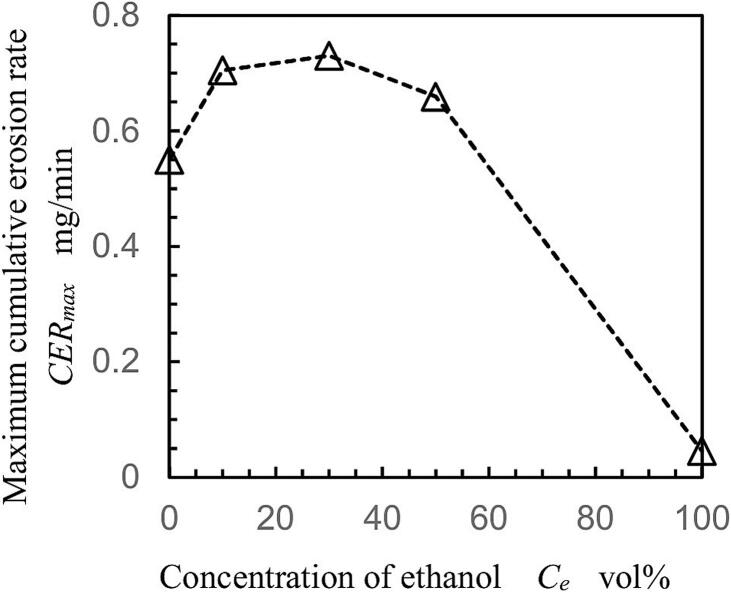
Maximum cumulative erosion rate (*CER*_max_) across varying concentrations of ethanol.

**Table C1:** Effect of flow rate on noise level (30 vol% ethanol aqueous solution).

			Condition A	Condition B
Upstream pressure of the Venturi tube	*p*_1_	[MPa]	3.4	3.4
Downstream pressure of the Venturi tube	*p*_2_	[MPa]	0.04	0.28
Pressure difference	*Δp* = *p*_1_ – *p*_2_	[MPa]	3.36	3.12
Flow rate	*Q*	[L/min]	1.895 ± 0.010	1.890 ± 0.004
Ratio of flow rate	*Q*_A_ / *Q*_B_	[–]	1.003 ± 0.006	
Cavitation number	*σ*	[–]	0.04	0.12
Flow coefficient	*c_f_*	[–]	0.879 ± 0.005	0.910 ± 0.002
Noise level	*N_L_*	[dB]	79.5 ± 0.7	90.3 ± 0.7
Normalized aggressive intensity of cavitation	*I_cav_’*	[–]	2.82 ± 0.12	33.37 ± 0.40

**Fig. C2:**
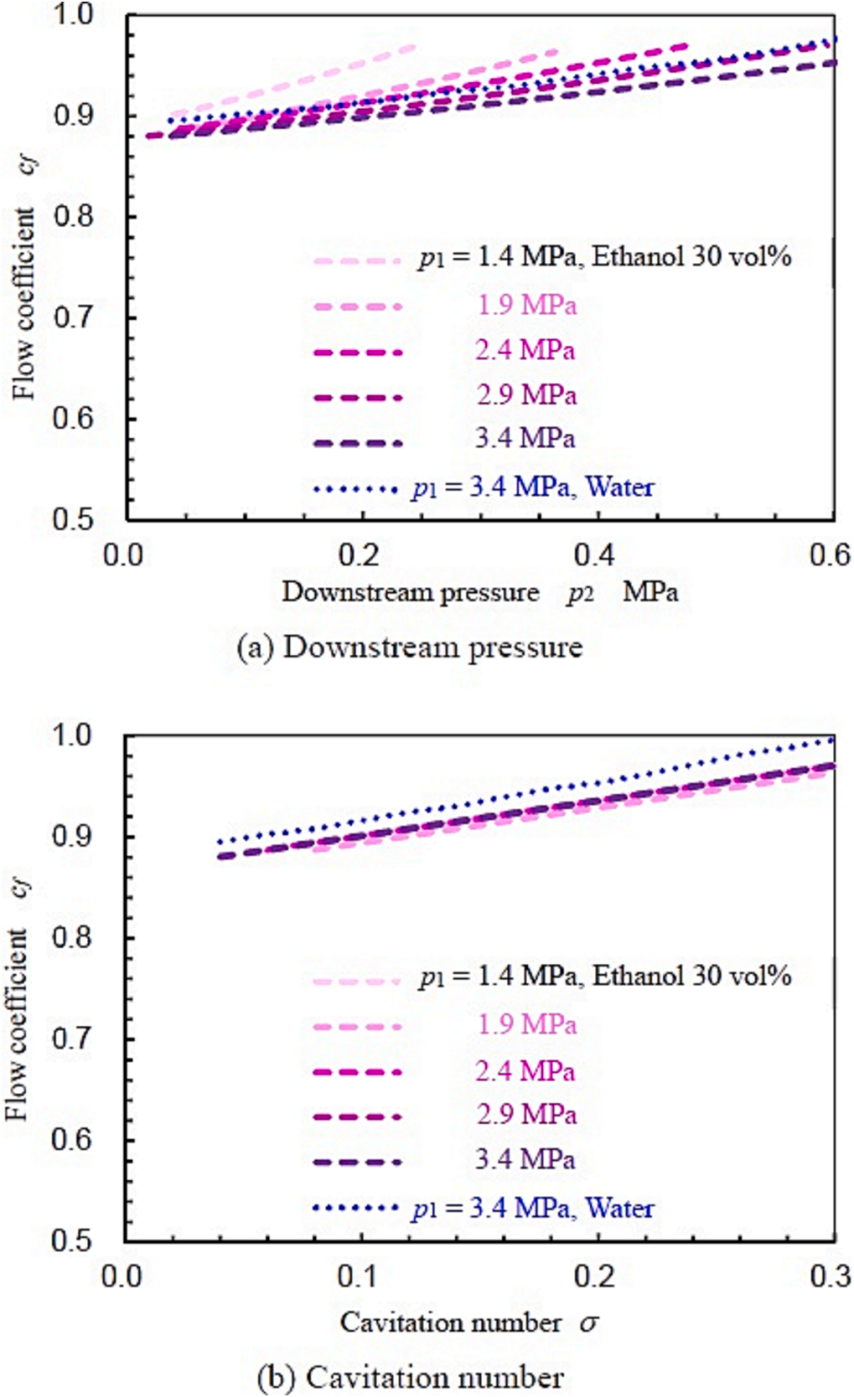
Flow coefficient as a function of downstream pressure and cavitation number

**Fig. D1:**
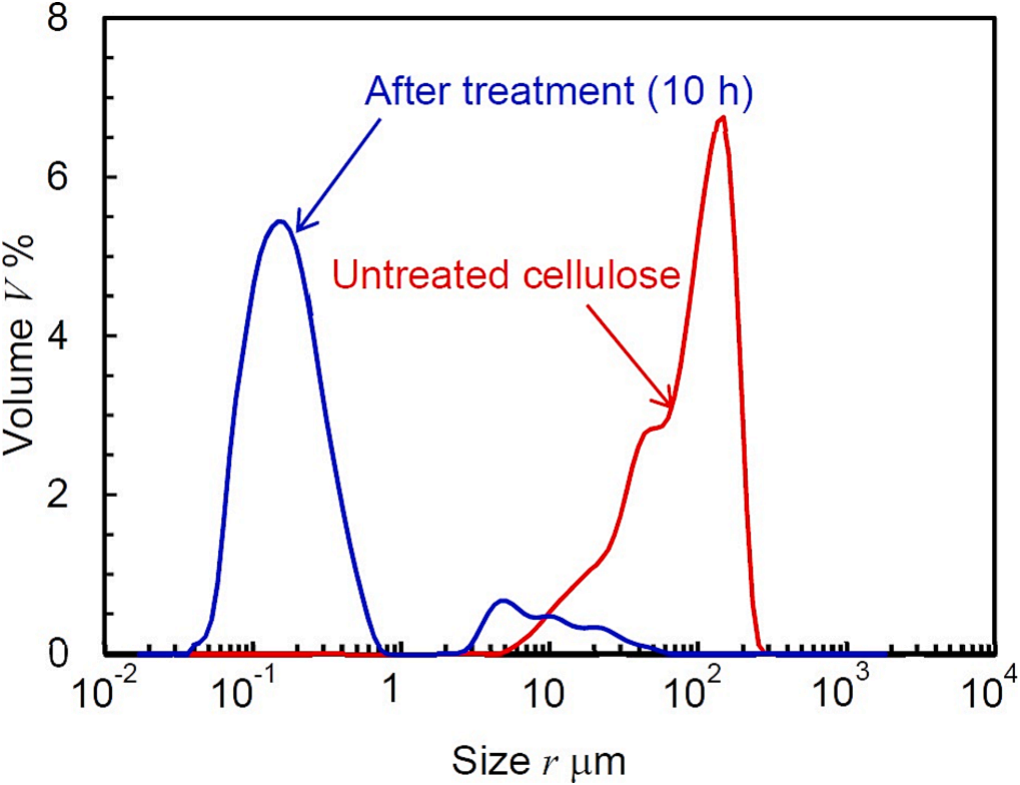
Particle size distributions of samples measured by particle size analyzer

**Fig. D2:**
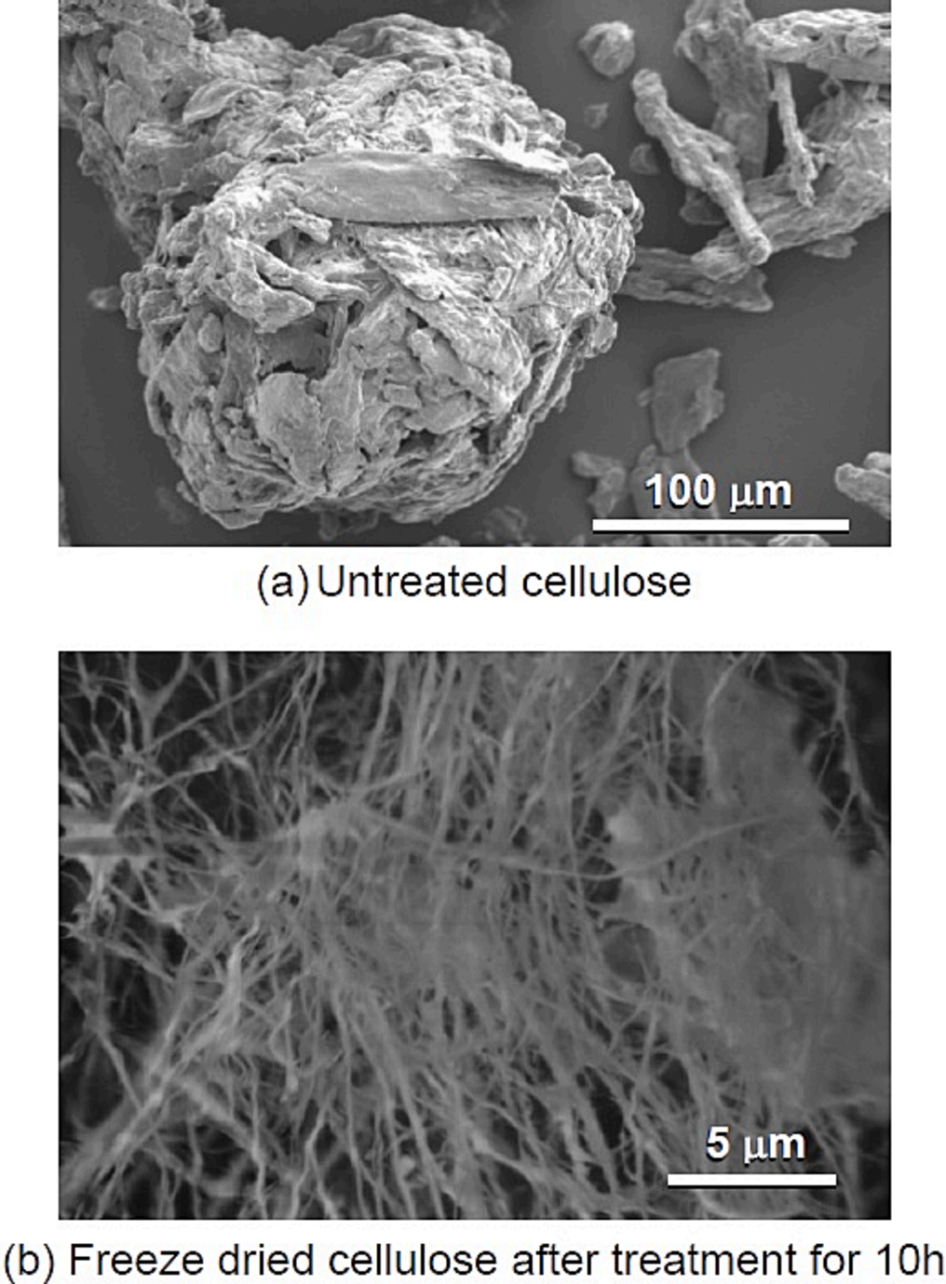
SEM images of untreated cellulose and cellulose treated by hydrodynamic cavitation

**Table D1:** Diameter of samples measured by particle size analyzer.

	Untreated cellulose	After treatment
Average diameter	100.6 ± 58.3 μm	1.619 ± 5.289 μm
Median diameter	98.23 μm	0.184 μm
Mode diameter	153.8 μm	0.155 μm

**Fig. E1:**
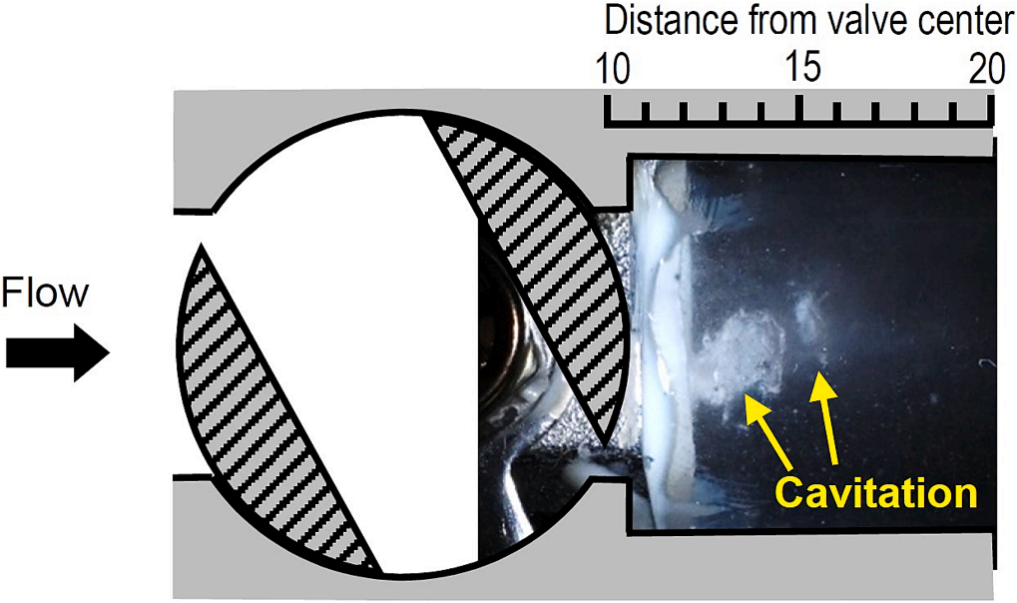
Typical aspect of cavitation arising in downstream of ball valve (Water)

**Fig. E2:**
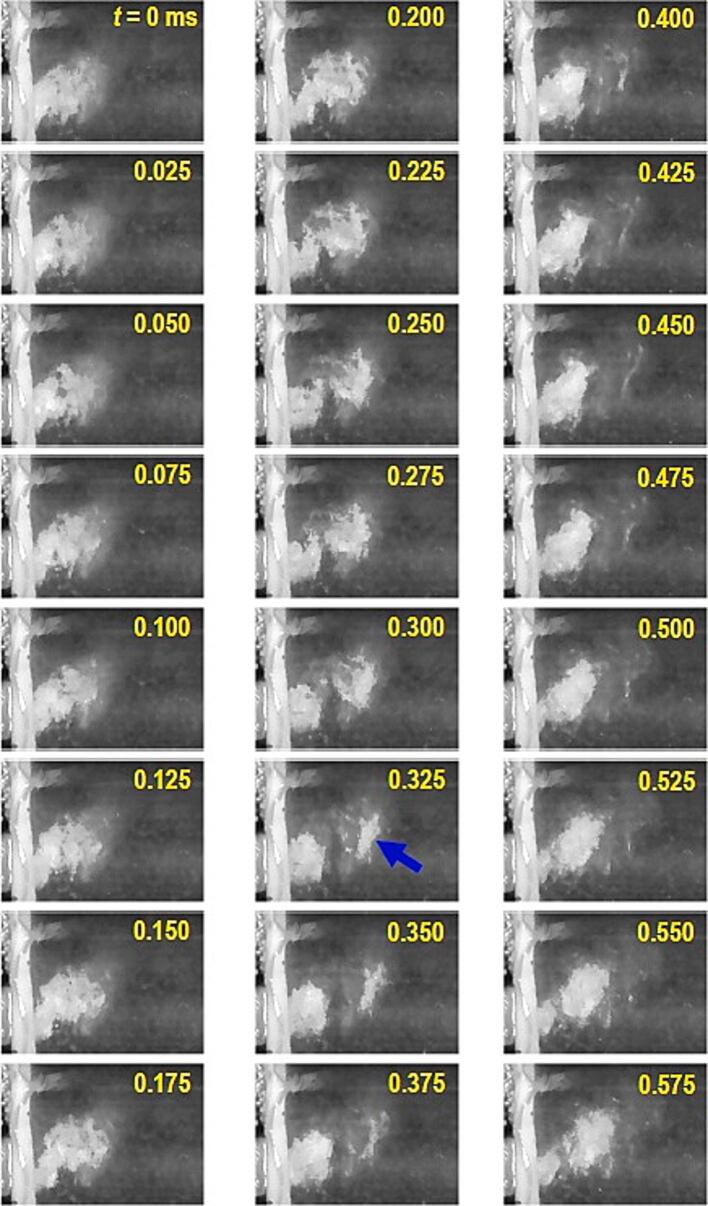
Aspect of cavitation arising in downstream of ball valve changing with time (Water)

**Fig. E3:**
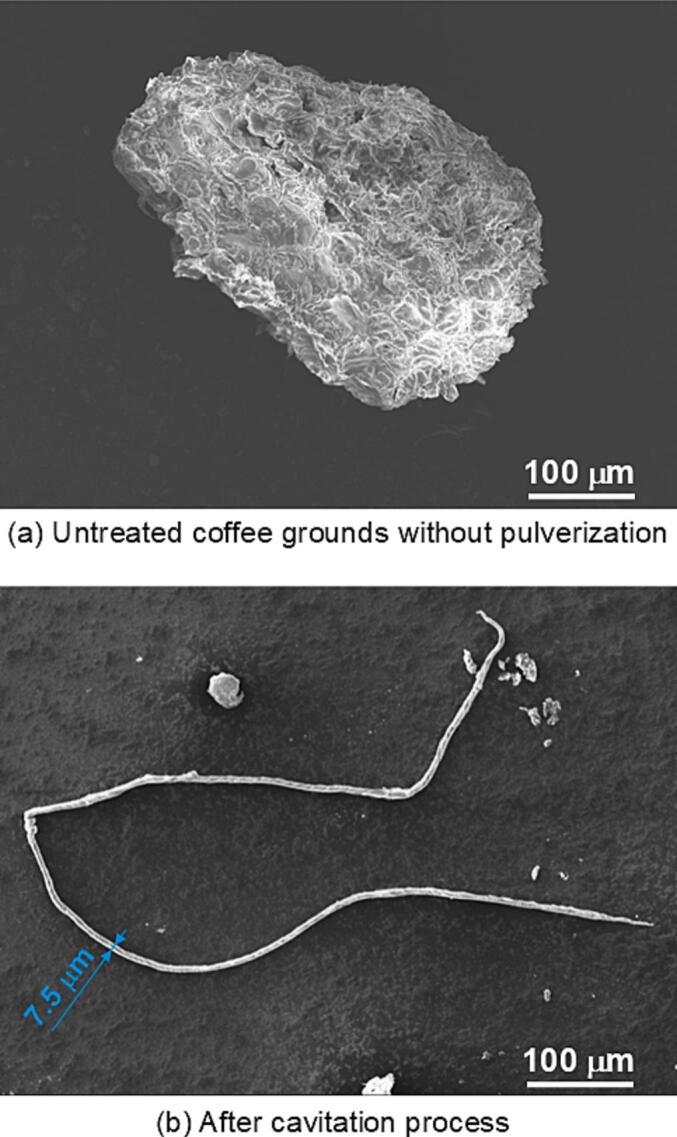
Production of microfibril and sub-micro crystals by hydrodynamic cavitation arising in ball valve

## Appendix A . Luminescence intensity changing with the concentration ratio of ethanol

Fig. A1 displays the luminescence intensity caused by hydrodynamic cavitation in a Venturi tube as a function of the downstream pressure (*p*_2_). The experimental setup and procedures align with those described in a previous study[99]. Luminescence intensity (*I_L_*) measurements were taken at ethanol concentrations (*C_e_*) of 0, 10, 20, 30, 40, and 50 vol%. According to the “*Materials*” section 2.1, the injection pressure, also known as the upstream pressure of the Venturi tube, was set at 0.5 MPa. The *I_L_* at *C_e_* values of 0, 10, 20, 30, 40, and 50 vol% each showed a peak in relation to *p*_2_. In Fig. A1, *I_L_* was normalized to the peak value at *C_e_* = 0 vol%, representing water, denoted as *I_L_*. Fig. A2 illustrates the maximum luminescence intensity (*I_Lmax_*) for each *C_e_*, indicating that *I_Lmax_* peaks at *C_e_* = 30 vol%. Notably, the *I_L_* of *C_e_* = 30 vol% reached its maximum at *p*_2_ = 0.14 MPa, and *I_Lmax_* of *C_e_* = 30 vol% was 2.1 times greater than that of water (*C*_e_ = 0 vol%.

## Appendix B . Cavitation erosion rate changing with the concentration ratio of ethanol

To explore the aggressive intensity of hydrodynamic cavitation under relatively high injection pressures, Fig. B1 depicts the change in mass loss over erosion time when exposed to a cavitating jet, using an ASTM G134 apparatus [101]. A cavitating jet, a type of hydrodynamic cavitation, is a submerged high-speed water jet that induces cavitation around itself. When materials are exposed to this jet, they experience mass loss due to cavitation. The cumulative erosion rate (*CER*) was defined as the mass loss divided by the erosion time, and the *CER* reached a maximum (*CER_max_*) at a specific erosion time [101]. Higher aggressive intensities of the cavitating jet correlated with higher *CER_max_*. A cavitation erosion test was employed to assess the aggressive intensity of the hydrodynamic cavitation. In this test, the injection pressure was set at 10 MPa, and the cavitation number (*σ*) was selected as 0.01485, as the peak aggressive intensity of the cavitating jet occurred at *σ* ≈ 0.014. Copper was used as the test material, and the liquid temperature was maintained at 303 K. As depicted in Fig. B1, the mass loss (*Δm*) increases with erosion time (*t_e_*). For a solution of 100 vol% ethanol, the slope of *Δm* versus *t_e_* was notably less steep compared to that for water (0 vol% ethanol). However, the slopes at ethanol concentrations of 10, 30, and 50 vol% were steeper than those for water. Fig. B2 demonstrates the variation of *CER_max_* with *C_e_*, showing a maximum at *C_e_* = 30 vol%.

## Appendix C . Effect of flow rate

In order to reveal the effect of flow rate *Q* on present cavitating flow through the Venturi tube, Fig. C1 illustrate *Q* changing with *p*_2_ for 30 vol% ethanol aqueous solution and water. Fig. C2 shows the flow coefficient *c_f_* as a function of *p*_2_ (Fig. C2 (a)) and *σ* (Fig. C2 (b)). When *p*_2_ was changed at constant *p*_1_, *Q* was approximately equivalent as shown in Fig. C1. As expected, *c_f_* at lower *p*_2_ was slightly smaller than *c_f_* at higher *p*_2_ as shown in Fig. C2 (a), as cavitating region was developed as shown in Fig. 7. The relationship between *p*_2_ and *c_f_* differs for each *p*_1_. On the other hand, the relationship between *σ* and *c_f_* of 30 vol% ethanol aqueous solution is almost on the same line as shown in Fig. C2 (b), even if *p*_1_ is different. Namely, the key parameter of the decrease of *c_f_* was *σ*. As an example, Table C1 shows *p*_1_, *p*_2_, *Δp*, *Q*, *σ*, *c_f_*, *N_L_* and *I_cav_*’ for conditions A and B. Condition A corresponds to lower *p*_2_ condition and condition B corresponds to *p*_2 max_ at which noise level *N_L_* had a maximum as shown in Fig. 4, Fig. 5. The *Q* was *Q*_A_ = 1.895 ± 0.010 L/min for condition A and *Q*_B_ = 1.890 ± 0.004 L/min for condition B. Thus, the ratio of flow rate *Q*_A_ / *Q*_B_ was 1.003 ± 0.006. Namely, *Q* at constat *p*_1_ were approximately equivalent. On the other hand, *c_f_* was 0.879 ± 0.005 for condition A and 0.910 ± 0.002 for condition B. Note that the decrease of *c_f_* was not so large at present condition of the used Venturi tube. Thus, the pressure difference *Δp* = *p*_1_ – *p*_2_ at condition A, i.e., 3.36 MPa was larger than that at condition B, i.e., 3.12 MPa. This is why *Q*_A_ and *Q*_B_ were approximately equivalent. Then, it can be concluded that the effect of *Q* on *N_L_* and *I_cav_*’ was negligible, as *I_cav_*’ at conditions B was 11.8 times larger than that at condition A.

## Appendix D . Comparison on size distribution between SEM observation and laser diffraction scattering method

In order to compare size distribution evaluated by a laser diffraction scattering method with SEM observation, Fig. D1 and Table D1 show the particle size distribution and diameters measured by a particle size analyzer (Beckman Coulter, Inc., Brea, CA, LS13 320) for untreated cellulose and the aqueous solution after hydrodynamic cavitation treatment. The sample was micro sized cellulose (Funacel II®, Funakoshi Co., Ltd, Tokyo, Japan). An aqueous solution containing 5 g of micro sized cellulose in 500 ml was treated hydrodynamic cavitation through Venturi tube at *p*_1_ = 3.1 MPa for 10 h. Fig. D2 shows aspect of untreated cellulose and a sample obtained by freeze-drying an aqueous solution after the hydrodynamic cavitation treatment observed by SEM. As shown in Fig. D1, Fig. D2, the hydrodynamic cavitation produces microfibrils from cellulose particles obviously. Regarding particle size distribution measured by the particle size analyzer, the particles size before treatment was 100 – 200 μm in diameter, and it became 100 – 200 nm in diameter by the treatment using the hydrodynamic cavitation. However, the SEM observations show that the diameter of the fine cellulose fibers after treatment was 10 – 20 nm. This is because the particle size analyzer assumed that the sample was spherical. Thus, at present experiment, SEM observation was chosen for the evaluation of fiber size.

## Appendix E . Production of cellulose microfibrils from coffee grounds without pulverization at high concentration ratio (23 %) using hydrodynamic cavitation

Fig. E1 shows a typical aspect of hydrodynamic cavitation arising in downstream of a ball valve, whose outer diameter of the ball was 12 mm and inner diameter of the hole in ball was 6.8 mm. The ball valve was used to generate cavitation, as the opening of the ball valve could be varied to avoid stacking due to large coffee grounds at the start of the process. The experimental setup was same as the present experiment which was shown in Fig. 1 except the Venturi tube. In Fig. E1, main flow direction was from left to right. The schematics of the ball valve was illustrated in Fig. E1. The upstream pressure was about 3 MPa. As the cavitating region generated by the ball valve was small, the downstream valve was opened. The opening of the ball valve was controlled by the upstream pressure gauge and the pump’s rotational speed to be equivalent to that of a Venturi tube with a 0.7 mm throat diameter. Fig. E2 reveals the aspect of cavitation arising in downstream of the ball valve observed by the high-speed video camera, whose recording speed was set at 40,000 fps. The typical vortex cavitation was indicated by the bule arrow in Fig. E2 at *t* = 0.325 ms. The ball valve can generate vortex cavitation which was observed in the Venturi tube. Namely, the hydrodynamic cavitation generated by the ball valve can be utilized for the production of cellulose microfibrils from coffee grounds without pulverization at high concentration ratio.

In order to demonstrate the production of cellulose microfibrils from coffee grounds without pulverization at high concentration ratio using hydrodynamic cavitation, Fig. E3 shows the SEM images of coffee grounds and microfibrils. 0.9 kg of the coffee grounds without pulverization were mixed into 3 L of the 30 vol% ethanol aqueous solution, and treated by the hydrodynamic cavitation arising in the ball valve for three hours. The concentration ratio of the coffee grounds was 23 %. The upstream pressure was about 3 MPa. The opening of the ball valve was controlled by the upstream pressure gauge and the pump’s rotational speed to be equivalent to that of a Venturi tube with a 0.7 mm throat diameter as same as cavitation observation. As shown in Fig. E3 (b), the micro-meter order fiber was obtained from the coffee grounds without pulverization. Namely, the high concentration ratio such as 23 % of coffee grounds without pulverization can be processed by the hydrodynamic cavitation generated by the ball valve, and the microfibrils were obtained.
